# Inhibition of primary breast tumor growth and metastasis using a neuropilin-1 transmembrane domain interfering peptide

**DOI:** 10.18632/oncotarget.10101

**Published:** 2016-06-16

**Authors:** Alexia Arpel, Coralie Gamper, Caroline Spenlé, Aurore Fernandez, Laurent Jacob, Nadège Baumlin, Patrice Laquerriere, Gertraud Orend, Gérard Crémel, Dominique Bagnard

**Affiliations:** ^1^ INSERM U 1109, MN3T Laboratory, Labex Medalis, Strasbourg University, Strasbourg, France; ^2^ CNRS UMR 7178, Institut Pluridisciplinaire Hubert Curien, Strasbourg University, Strasbourg, France

**Keywords:** neuropilin-1, breast cancer, metastasis, treatment, peptide

## Abstract

The transmembrane domains (TMD) in membrane receptors play a key role in cell signaling. As previously shown by us a peptide targeting the TMD of neuropilin-1 (MTP-NRP1), blocks cell proliferation, cell migration and angiogenesis *in vitro*, and decreases glioblastoma growth *in vivo*. We now explored the clinical potential of MTP-NRP1 on breast cancer models and demonstrate that MTP-NRP1 blocks proliferation of several breast cancer lines including the MDA-MB-231, a triple negative human breast cancer cell line. In models with long term *in vivo* administration of the peptide, MTP-NRP1 not only reduced tumor volume but also decreased number and size of breast cancer metastases. Strikingly, treating mice before tumors developed protected from metastasis establishment/formation. Overall, our results report that targeting the TMD of NRP1 in breast cancer is a potent new strategy to fight against breast cancer and related metastasis.

## INTRODUCTION

In spite of broad achievements in early breast cancer diagnosis, death due to breast cancer and related metastases remains a sobering fact [1, 2]. This indicates the need to develop new strategies and therapeutic tools with effective anti-metastatic properties. To address this need, we tested a novel strategy inhibiting the recently identified breast cancer target neuropilin-1 (NRP1), a membrane receptor involved in nervous system development and angiogenesis [3]. Mounting evidence now indicates a crucial role in breast cancer tumorigenesis and metastasis. NRP1 promotes breast cancer cell survival [4, 5] and different studies showed a role in cell migration and metastasis [6–8]. Consistently, the expression of NRP1 in human breast cancer tissue [7, 9, 10] negatively correlates with patient survival [7, 9]. Barr and co-workers demonstrated in 2005 [11] that a peptide targeting the VEGF165-binding site of NRP1, antagonises the autocrine anti-apoptotic effects of VEGF in cultured 4T1 and MDA-MB-231 breast carcinoma cells. Moreover, the group of Perret and colleagues described that a heptapeptide also inhibiting VEGF binding to NRP1 reduced tumor volume, blood vessel density in orthotopic mammary MDA-MB-231 tumors [12]. We had previously identified a peptide that antagonizes activities of the transmembrane domain in NRP1 (Membrane Targeting Peptide NRP1, MTP-NRP1). This peptide exhibited *in vitro* and *in vivo* anti-proliferative, anti-migratory and anti-angiogenic properties [13, 14] blocking glioblastoma growth. In the present study, we decided to explore whether targeting NRP1 with our peptide potentially is also suitable for blocking breast cancer growth and metastasis. Because of the lack of targeted therapy for triple negative breast cancer, we focused our study on the aggressive human breast cancer line MDA-MB-231 (Estrogen Receptor ER^−^, Progesterone receptor PR^−^, HER2^−^) recapitulating this unfavorable clinical context. We found that long term tri-weekly intraperitoneal administration of MTP-NRP1 significantly improved the overall survival of mice compared to vehicle-treated controls. This benefit could be attributed to reduced primary tumor growth as assessed by bioluminescence *in vivo* imaging and the RECIST criteria [15, 16]. We also report an anti-metastatic effect of MTP-NRP1 preventing establishment and growth of metastases. Hence, we provide data demonstrating the protective effect of MTP-NRP1 as it reduced metastasis formation and growth when administrated before grafting of tumor cells. Overall, our data not only validate the use of peptides antagonizing NRP1 as very powerful approach to fight triple negative breast cancer growth and metastasis, but also exemplify that drugs targeting the TMD of membrane receptors offer a convincing alternative to conventional drugs targeting extra- or intracellular domains.

## RESULTS

### MTP-NRP1 inhibits proliferation of 4T1 murine breast cancer cells

**Figure 1:**
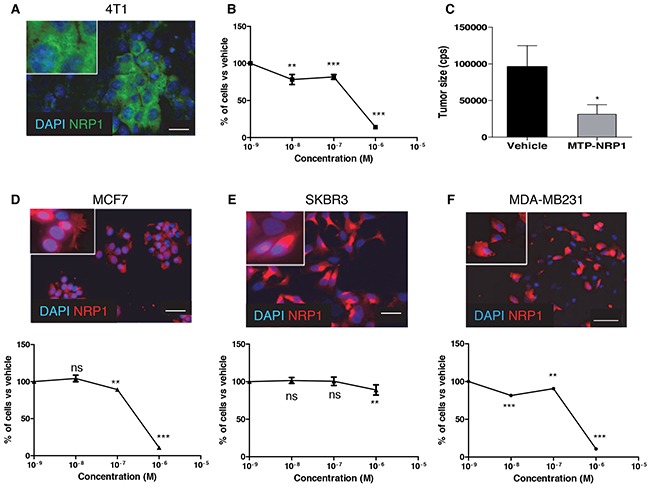
MTP-NRP1 inhibits breast tumor cell proliferation **A.** Expression of NRP1 in murine 4T1 cell line. **B.** MTT assay demonstrating the *in vitro* anti-proliferative activity of MTP-NRP1 peptide. **C.** Size of 4T1-derived subcutaneous tumors after 3 weeks every 3 days IP administration of MTP-NRP1 determined with bioluminescence. (* p < 0.05, Mann Whitney test). **D.** Expression of NRP1 and anti-proliferative activity of MTP-NRP1 on human MCF7 breast cancer cells. **E.** Expression of NRP1 and anti-proliferative activity of MTP-NRP1 on human SKBR3 breast cancer cells. **F.** Expression of NRP1 and anti-proliferative activity of MTP-NRP1 on human MDA-MB-231 breast cancer cells. (ns: not significant, ** p < 0.01 and *** p < 0.001, Mann Whitney test).

The transmembrane domain of NRP1 is 100% conserved across mammalian species thereby simplifying preclinical validation. To address the biological activity of MTP-NRP1 in breast cancer we first focused on a murine setting to evaluate the peptide in an immunocompetent mouse model. To this end, we performed a proliferation MTT assay on murine 4T1 cells expressing NRP1 (Figure 1A). We found a significant dose-dependent reduction of cell proliferation starting at a concentration of the peptide of 10^−8^M exhibiting a maximal effect at 10^−6^ M. We then produced subcutaneous tumors in Balb/C mice by grafting 4T1 cells. MTP-NRP1 was administrated for a period of three weeks every three days (1 μg/kg) when tumors reached a volume of 200 mm^3^. As seen in Figure 1C, the tumor size was 67% reduced in the MTP-NRP1 treated group compared to the vehicle receiving group. This demonstrated the anti-tumor effect of MTP-NRP1 in a syngenic model.

### MTP-NRP1 inhibits proliferation of human breast cancer cells

**Figure 2:**
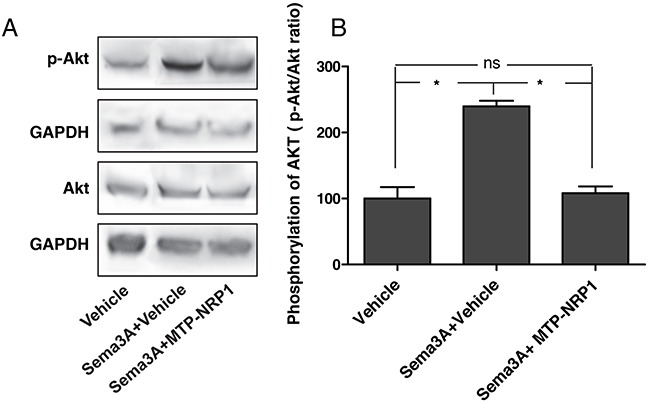
MTP-NRP1 inhibits Sema3A-induced phospho-AKT **A.** Representative western blots showing the induction of AKT phosphorylation upon treatments with 200ng/ml Sema3A and with or without MTP-NRP1. Akt and p-AKT expression levels were normalized with GAPDH. **B.** Quantitative analysis of 3 independent experiments showing the relative expression of p-AKT/AKT in the different experimental conditions. (*= p<0.05, ns: not significant; Mann Whitney test).

To address the biological activity of MTP-NRP1 on human breast cancer cells we next performed a proliferation MTT assay on three different NRP1 expressing cell lines, MCF7, SKBR3 and MDA-MB-231 (see Figure 1D-1F showing the expression at protein level). Q-RTPCR analysis confirmed the expression of NRP1 and showed similar amounts of mRNA for all cell lines (see Supplementary Figure S1). Cells were treated with increasing doses of MTP-NRP1 ranging from 10^−8^M to 10^−6^M for 24 hours. MTP-NRP1 induced a significant reduction of cell numbers as from 10^−8^M in MDA-MB-231 (−11% up to −89% at 10^−6^M), 10^−7^M in MCF7 cells (−12% up to −90% at 10^−6^M) and 10^−6^M in SKBR3 (−12%). Higher concentrations were not possible to test because of a non-specific toxicity of the vehicle. Because the triple negative MDA-MB-231 cells are highly metastatic and represent an important therapeutic challenge we decided to focus on this cell line for in vivo experiments. Strikingly, the addition of MTP-NRP1 (10^−6^M) fully blocked the Sema3A-induced phosphorylation of AKT (Figure 2). Because of the important role of AKT in the metastatic process this result strengthened the need to investigate the therapeutic potential of MTP-NRP1 in vivo. Considering reported cases of toxicity with anti-NRP1 antibodies we decided to use the 10^−7^ M as a compromise between efficacy and low risk of toxicity. While not producing the maximal anti-proliferative effect in vitro, this concentration was indeed previously proven efficient and safe when treating brain tumors [13]. Hence, we monitored tumor growth by bioluminescence quantification at week 2, 5, 9 and 13 upon injection of luciferase expressing MDA-MB-231 by a life imaging system (NightOwl, Berthold). Initial experimental conditions were standardized by establishing groups of similar cumulated bioluminescence two weeks after grafting of 10^6^ cells in the mouse mammary fat pad (average bioluminescence in the vehicle group being 4147 cps and 4189 cps in the MTP-NRP1 group (p = 0.9, Mann Whitney). Mice were treated three times a week by intra-peritoneal injection of either the vehicle (LDS, 72 μM) or MTP-NRP1 (10^−7^M). A total number of 12 animals composed each group. Figure 2A is showing representative examples of the orthotopic tumors detected in the control and the MTP-NRP1 treated groups over time. To analyze extensively the response of each mouse to the treatment a waterfall plot of best response was applied by using the bioluminescence increase between week 9 and 13. This analysis revealed that 100% of the MTP-NRP1 treated animals responded to the treatment with 25% of SD (Stable Disease, < −30% growth decrease compared to averaged growth of control tumors) and 75% responded with PR (Partial Response >-30% growth decrease compared to averaged growth of control tumors) including two individuals above or equal to 90% decrease of tumor volume expansion (Figure 2B). This part of the study demonstrated that blocking NRP1 in triple-negative breast tumors significantly blocks tumor growth in all treated mice.

**Figure 3:**
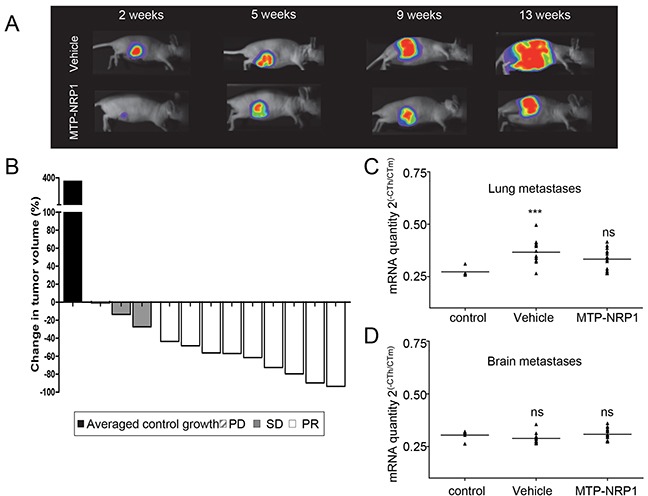
MTP-NRP1 inhibits primary breast tumor growth *in vivo* **A.** Representative examples of the orthotopic tumors (MDA-MB-231 cells) detected in the control or the MTP-NRP1 group treated with 1 μg/kg three times a week over time (2, 5, 9 and 13 weeks). **B.** Demonstration of the inhibitory effect of MTP-NRP1 on primary tumor growth between week 9 and 13 of treatment. The Waterfall graph represents the percent change in tumor volume of individual treated animals (grey and white bars, n=12) compared to the averaged tumor volume increased determined in the control group (dark bar, n=12). This demonstrates that 100% of the treated animals responded to MTP-NRP1 treatment including 25% with stable disease (SD grey bars) and 75% with partial response (PR white bars). SD (Stable Disease, < −30% growth decrease compared to averaged growth of control tumors), PR (Partial Response > −30% growth decrease compared to averaged growth of control tumors) **C.** RT-QPCR analysis of human specific HMBS mRNA content in the lung to reflect metastasis colonization. Results are mRNA quantity compared to the corresponding mouse specific HMBS housekeeping gene and expressed as 2^(−CT_Hs_HBMS/CT_Ms_HBMS)^. Statistical analysis was done by comparing mRNA content with background signal determined in the lung of control mice without primary tumors (ns= not significant, ** p < 0.01, Mann Whitney test). **D.** Similar analysis was conducted in the brain. However, no significant human specific HMBS signal was detected in this organ.

The duration of our protocol was not sufficient to detect metastasis in any organs at bioluminescence or histological level. Therefore, we applied a more sensitive approach by measuring the human specific mRNA coding for the human specific HBMS housekeeping gene and compared expression to the corresponding mouse specific HBMS gene transcript in vehicle or MTP-NRP1 treated mice. This approach allowed us to detect lung metastases in the vehicle group, yet not in other organs such as the brain (Figure 3). Strikingly, we found that MTP-NRP1 significantly reduced lung metastasis issued from the primary tumor almost to background level. Due to insufficient production of metastases we decided to use a different model to evaluate the anti-metastatic effect of MTP-NRP1.

### MTP-NRP1 exhibits anti-metastatic properties

**Figure 4:**
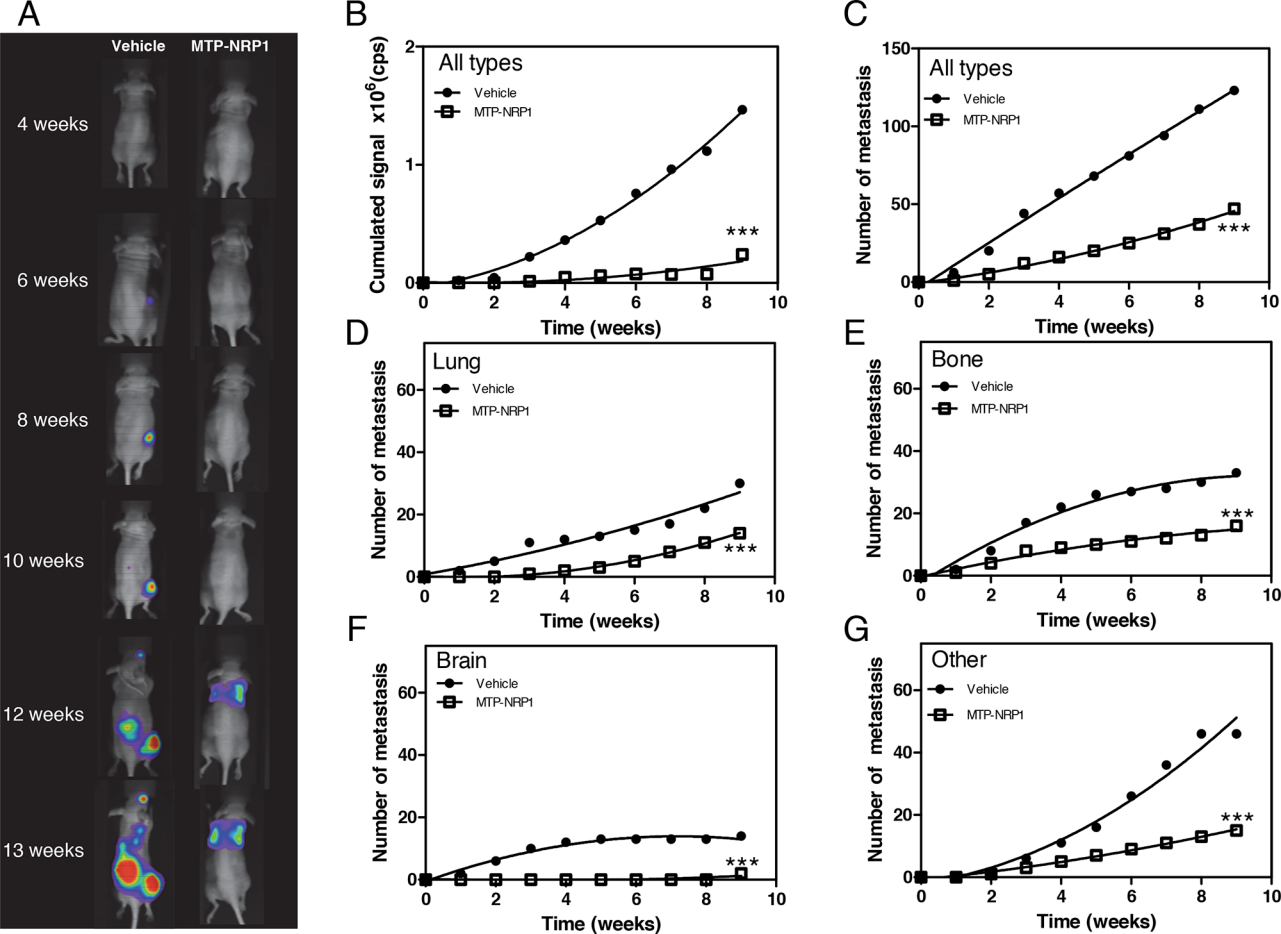
MTP-NRP1 exhibits anti-metastatic properties **A.** Representative examples of the metastases (MDA-MB-231 cells) detected in the vehicle and MTP-NRP1 treated groups over time (4, 6, 8, 10, 12 and 13 weeks). **B.** Cumulated bioluminescence signal obtained in vehicle (control) and MTP-NRP1 treated groups over time. **C.** Cumulated number of metastases in entire animals over time. Detailed analysis of cumulated number of metastasis in lung **D.**, bone **E.**, brain **F.** and other sites **G.** in control and MTP-NRP1 treated animals. (*** p < 0.001, Extra sum-of-squares F test).

The occurrence of lung metastasis is a crucial step in breast cancer progression and is linked to the disease-associated death [17]. Thus, we decided to monitor whether MTP-NRP1 would impede breast cancer metastasis development and progression. Mice were here again treated with 1.5 μg/kg of MTP-NRP1 or with the vehicle (LDS, 72μM). Treatment was started 2 days post-intracardiac grafting of 10^5^ MDA-MB-231 cells in the left ventricle. Figure 4A is exhibiting representative examples of mice developing metastases over time in the two experimental groups. The location of metastasis was confirmed at histological level in pilot studies to validate accuracy of the method (see Supplementary Figure S2). Strikingly, the quantification of the cumulated bioluminescent signal showed that MTP-NRP1 dramatically reduced the number (−62%, Figure 4C) and size (−83%, Figure 4B) of the arising metastases. Noteworthy, when further addressing the sites of metastasis (in the lung, bone and brain, the major metastatic sites in human breast cancer) we found that the number of metastasis decreased significantly in all analyzed sites upon treatment with MTP-NRP1 (Figure 4D–4G).

### MTP-NRP1 improves overall survival

**Figure 5:**
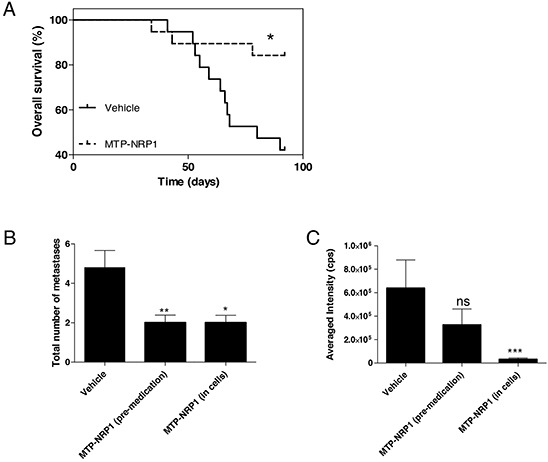
MTP-NRP1 improves overall survival and exhibits protective effect against metastasis **A.** Kaplan Meier survival curve demonstrating a significant increased survival of the treated MTP-NRP1 animals compared to the control animals. (* p < 0.05, Log Rank test). **B.** Total number of metastases determined in animals that had received IP administration of MTP-NRP1 for 3 days before intracardiac grafting (“pre-medication”) or that were grafted with cells (all MDA-MB-231) pre-incubated with the peptide (“in cells”). **C.** Averaged intensity of bioluminescence (cps) emitted by metastases that were able to grow in the different experimental groups. (ns= not significant, * p < 0.05, ** p < 0.01, *** p < 0.001, Mann Whitney test).

To finally address the therapeutic benefit of MTP-NRP1 we also monitored overall survival (OS) of mice during the whole protocol. Consistently, the high response rate of mice to MTP-NRP1, both in terms of reduced primary tumor volume and number of metastasis, translated into a significant survival benefit (+ 24.7% of the mean survival, p = 0.0109 compared to vehicle, Log-ranked test), a survival benefit also exemplified by a 41.5% increase of the median survival (Figure 5). It is important to note that this protocol lasted for 92 days during which only 16% of the mice died in the MTP-NRP1 group while death reached 58% in the control group. This long term three weekly injection mimicking chronic application provided the possibility to evaluate a potential toxicity of MTP-NRP1 arising after longer term treatment. Blood samples were collected from the cardiac cavity for analysis before sacrifice of animals of both the orthotopic and the systemic metastasis model. In all cases, MTP-NRP1 did not worsen biological parameters of mice including renal, hepatic and cardiac markers. A seen in Supplementary Table S1 and S2, we rather observed a significant lower level of Lactate Dehydrogenase (LDH) reflecting better function of organs in the treated group. Interestingly, the level of platelets was significantly lower in the control group compared to MTP-NRP1 treated mice. Because a transient reduction of platelets in patients treated with an anti-NRP1 antibody was one of the most common (67% of the patients) reported adverse effect in a phase 1 clinical study, this result further demonstrated the good body tolerance for MTP-NRP1.

### MTP-NRP1 treatment prevents metastasis formation

The anti-metastatic effect of MTP-NRP1 on primary tumors and induced lung metastases together with the good tolerance of the peptide prompted us to test whether protective treatment of mice by the peptide before grafting the tumor cells potentially was beneficial. We produced metastases by engrafting MDA-MB-231 cells through intra-cardiac injections after 3 days of continuous pre-medication of mice with MTP-NRP1 or by pre-incubating the tumor cells with the peptide 1h before engraftment. We found that both pre-treatments strongly reduced the occurrence of metastases by roughly 60% when determined 3 weeks post grafting (Figure 5B). This result suggested that blocking NRP1 is able to reduce metastatic colonization. Over the 3 weeks period of survey, the few metastases that had developed in mice receiving premedication were growing similarly well as the control group (Figure 5C). However, those metastases originating from cells that had been pre-incubated with the MTP-NRP1 peptide were 95% smaller (p=0.0006 Mann Whitney test, Figure 5C). These results demonstrated that pre-medication has a beneficial impact on metastasis development and growth. In addition, our results clearly demonstrated a NRP1 cell autonomous metastatic mechanism.

## DISCUSSION

The action mechanism of MTP-NRP1 has been well documented and is known to mainly relate to the interference of receptor dimerization [13, 14, 18, 19]. Here, we have shown an anti-proliferative property of MTP-NRP1 in one murine cell line and three different human breast cancer cell lines. The efficacy was stronger in MCF7 and MDA-MB-231 but modest in SKBR3 cells. This milder effect in SKBR3 may be due to the overexpression of HER2 providing a dominant pathway for cell proliferation [20]. Nonetheless, MTP-NRP1 appeared as a very potent inhibitor of primary breast tumor growth generated with 4T1 cells grafted in immunocompetent Balb/C mice. The demonstration of the clinical potential of MTP-NRP1 was confirmed by producing orthotopic breast tumors in nude mice grafted with human MDA-MB-231 cells in the mammary fat pad. When using objective individual tumor growth parameters adapted from the RECIST criteria [15, 16], we found that 100% of mice responded to the treatment. Furthermore, MTP-NRP1 induced a significant decrease of lung metastasis arising from the primary tumors. This anti-metastatic effect was further explored in the intra-cardiac grafting assay that showed both a reduction in the number and in the size of metastasis in all three major sites observed in human, the bone, lung and brain. The anti-tumor effect is obtained with a very low dosage of 1 μg/kg of MTP-NRP1. This is in accordance with our previous work that showed successful inhibition of glioma growth *in vivo* with the same concentration of MTP-NRP1. However, in the case of breast cancer cells, the maximal effect was obtained in vitro with 10^−6^M, a concentration similar to the one employed for MTP-NeuNT peptide targeting the TMD of the NeuNT receptor [21]. Based on our results future studies can be launched to further explore other parameters such as higher MTP-NRP1 concentrations to reach the maximal anti-tumor effects (optimal dosage, maximum tolerated dose), best therapeutic scheme (duration of treatment, therapeutic window) and potential combinations with other anti-breast cancer drugs. Future PK/PD analyses are necessary that currently are not yet possible due to the lack of tools to detect and measure hydrophobic peptides. Importantly, based on blood analysis after the long period of treatment (13 weeks), the MTP-NRP1 peptide did not show any toxicity. Although we did not reach the maximal tolerated dose here, similar experiments had been done in the glioblastoma model where no toxicity was seen in any tested organ. Noteworthy, no cutaneous lesions were observed on all mice around the intra-peritoneal injection site of the drug. This suggested the lack of peptide accumulation at the injection site avoiding a risk of local toxic side effect. A phase I study of the human monoclonal anti-NRP1 antibody MNRP11685A showed good tolerance when administrated as a single agent. This study only reported cases of transient platelet count reductions without severe impact. We did not observed such a phenomenon in our study thereby suggesting that MTP-NRP1 is extremely well tolerated with the low yet effective dosage we used. It will be interesting to further evaluate the tolerance in future studies dedicated to the identification of the best dosage in stand alone or in combination with other drugs.

Finally, pre-medication using MTP-NRP1 showed a clear reduction of metastasis occurrence/appearance. When administrating the peptide to the mice before grafting tumor cells, the number of metastatic events dropped suggesting that the seeding capacity of the cells is affected when blocking NRP1 in the microenvironment. However, this pretreatment was inefficient on cells that had succeeded extravasation from the blood stream. The cells were able to soil [22] and develop metastases demonstrating that the pre-medication preferentially impacted on tumor cells before breaching blood vessels to enter the lung parenchymal tissue. When the cells were pre-incubated with the peptide, thereby blocking NRP1 cell–autonomous signaling pathways, we found both a reduction of the number and size of metastases. This illustrates that NRP1 is involved in both cell autonomous and non-cell-autonomous mechanisms controlling the metastatic process.

Targeting the TMD of bitopic receptors such as NRP1 provides a new exquisite therapeutic tool. Previous work demonstrated a crucial role of the TMD of NRP1 and suggested that inhibition may present a therapeutic potential in glioma treatment [13, 14]. This strategy has been now validated for another type of cancer, breast cancer and its related metastases. Our results clearly revealed that a peptide mimicking the TMD of NRP1 decreases both the size and the number of breast metastasis translating into a marked improvement of survival. Importantly, this therapeutic benefit was achieved by using the drug in a micromolar dosage with no apparent toxicity. Hence, targeting the TMD of NRP1, with a peptide mimicking its TMD could be a future potent drug in breast cancer therapy particularly to prevent metastasis formation by administrating long term low dose MTP-NRP1 upon reduction or surgical removal of the primary tumor.

## MATERIALS AND METHODS

### Cell culture

The murine 4T1 cells and human epithelial breast adenocarcinoma derived from pleural effusion MDA-MB-231 (ER-, PR-, HER2-), MCF7 (ER+, PR+, HER2+), SKBR3 (ER-, PR-, HER2+) cells were grown in Dulbecco's modified Eagle medium (DMEM, GIBCO) and HUVEC (Human Umbilical Vein Endothelial Cells, Promo Cell) cells were cultured in Endothelial Cell Growth Medium (Promo Cell). MDA-MB-231 cells were purchased from ECACC (92020424), HUVEC cells were purchased from Promo Cell (C-12200), MCF7 and SKBR3 cells were obtained from our institute collection. All culture media were supplemented with 10% fetal calf serum (FCS) (Gibco), 100 I.U./ml penicillin, 100 μg/ml streptomycin (Sigma), and cultured at 37°C, 5% CO_2_. Cells were detached with trypsin-EDTA (0.05 % trypsin, 1X EDTA), spin down and split regularly up to 40 passages before new stocks were thawed.

### Peptides

Peptides have been synthesized by Peptide Specialty Laboratories GmbH using automatic peptide synthesis (Fmoc chemistry). The peptide corresponding to the TM sequence of NRP1: ILITIIAMSALGVLLGAVCGVVLYRKR is referred as MTP-NRP1. Peptides purity estimated by RP-HPLC was more than 95% according to manufacturer indication. Peptides were solubilized in LDS (Lithium Dodecyl Sulfate, 72 mM for stock solution) as previously described [13, 14].

### RT-QPCR

mRNA was extracted with TriReagent solution according to manufacturer's instruction (Molecular Research Center Inc., Euromedex). mRNA was treated with DNaseI (Invitrogen) and reverse transcribed using the High Capacity cDNA RT Kit (Life Technologies). Quantitative reverse transcriptase polymerase chain reaction (RTQ-PCR) was performed using the Power SYBR Green PCR Master Mix or TaqMan Gene Expression Master Mix (Life Technologies) using the 7500 Real time PCR System (Life Technologies) following the manufacturer's protocol. We used human specific Hs_HMBS (QT 00014462, Quiagen) and mouse specific Ms_HBMS (QT00494130, Quiagen) to quantify lung or brain metastasis contents. Samples were analyzed using 2μl cDNA. Calculation were effectuated as the following: Δct(MDA-MB-231^HBMS^) = ct (Hs_HBMS) – ct (Ms_HBMS), mRNA quantity = 2^(−ΔctMDA-MB-231HBMS)^.

### Western blot analysis

MDA-MB-231 cells were seeded on 6-well plates overnight, and then treated with 200ng/ml Sema3A (R&D 1250-S3) for 30 minutes prior to addition of 10^−6^M of MTP-NRP1 for one additional hour. Proteins were extracted in Laemmli buffer complemented with proteinases (Roche, #11836145001) and phosphatases (5mM of Na ortho-vanadate) inhibitors. They were loaded on a 4-15% gradient gel (Biorad). Antibodies for Akt, phospho Akt (Cell Signaling #4060 and #9272 respectively), GAPDH (Santa Cruz #SC-20357) and their respective rabbit and goat–HRP secondary antibodies (GE Healthcare) were used. Revelation was performed using Pierce™ ECL Plus Western Blotting Substrate and the PXi imager apparatus (Syngene Bio Imaging, UK). Quantification was done with Image J software.

### Immunocytochemistry

Cells were grown on sterile glass cover slips for one day before immunofluorescence staining. The cells were fixed with freshly made fixative 4% formaldehyde (FA) for 10 minutes. The samples were gently rinsed with PBS (1 wash for 10 minutes) before adding fetal calf serum blocking solution (FCS 5%) for a minimum of 30 minutes. Cells were permeabilized using 1x PBS with 0.1% Tween20 for 5 minutes. Anti-neuropilin-1 (sc-5541; Santa Cruz Biotechnology) diluted in 5% FCS-PBS was added to the cell over night at room temperature. After thorough wash (3 washes of 5 minutes) appropriate secondary antibody (goat anti-rabbit, Nordic Immunology GAM/Fab/TRITC diluted 1/1000 from stock) was added at room temperature for 90 minutes. After washing in PBS, the cell nuclei were stained with DAPI (4′,6-diamidino-2-phenylindole, 1/30 000 in water) for 10 minutes. Glass coverslips were finally mounted on microscopy glass slides using a polymerization medium (FluorSave reagent, Calbiochem-Merck, cat#345789).

### Cell proliferation

*In vitro* cell proliferation was monitored using MTT (3-(4,5-Dimethylthiazol-2-yl)-2,5-diphenyl tetrazolium bromide) proliferation assay according to manufacturer's instruction (Sigma, M2128, USA). Cells were seeded at a density of 10 000 cells per well in a 96 well plate before incubation with increasing peptide concentration (ranging from 10^−9^M to 10^−6^M) or corresponding vehicle increasing concentration (LDS, ranging from 0.72 μM to 720 μM). After 24h incubation, the culture media were removed from the wells and 100 μl of MTT dye freshly diluted (to 1/50 in GBSS) from stock solution (5 mg/ml) was added to each well for 4h. After this incubation period, isopropanol (100 μl) was added to the MTT solution in each well before reading the optical density at 570 nm using a microplate reader spectrophotometer (EL800, Bio Tek Instruments). For cell fluorescence acquisition, images were acquired with the fluorescence Zeiss Imager Z2 equipped with HXP 120W lamp and structured light ApoTome (Zeiss) system.

### Orthotopic grafting of cells in the mouse mammary fat pad

Nude mice (8 weeks of age) were anesthetized (initially 3% isoflurane with air/O_2_ mix, then animals were kept under anaesthesia with 1.5% Isoflurane with air/O_2_ mix). A cutaneous incision up to the sternum was completed, followed by another angled lateral incision from the initial one towards the posterior leg. The blood vessel emerging between these fat pads was cauterised (Electric cauterizer (FST No 18000-00). Then the needle (BD Microlance; 22G ¼ - Nr 12; 0,7 × 30 mm, REF 300900) of the syringe containing 10^6^ cells in 50μl of PBS was inserted in the mammary fat pad from the external side up to the lymph node and the cells were injected behind the lymph node within the mammary fat pad. Skins were then aligned and sutured. The mouse was monitored until it was awakened from the procedure and was moving around the cage normally and then observed on a daily basis until sacrifice. For bioluminescence detection, IP injection of 100μl of a luciferin solution at 30mg/ml was completed on a weekly basis for each mouse. Acquisition was operated for 5min using a live imager (NightOwl, Berthold). All treatments were administrated by IP injection of 100μl solutions containing 72μM LDS (control group) or 1 μg/kg MTP-NRP1. Injections were performed every 3 days once the tumors reached a volume of 200 mm^3^. The percent change in bioluminescence intensity at week 9 and 13 was used to quantify response. PD (Progression disease, >20% increase compared to averaged growth of control tumors), SD (Stable Disease, < −30% growth decrease compared to averaged growth of control tumors), PR (Partial Response > −30% growth decrease compared to averaged growth of control tumors), and CR (Complete response, 100% growth decrease compared to averaged growth of control tumors) were defined as per RECIST criteria.

### Intra-cardiac grafting model

Cells were detached with Versene (EDTA solution used in order to assess a gentle non-enzymatic cell dissociation), washed and counted for 10^5^ in 100μl of PBS before injection into the left ventricle of nude mice (8 weeks old) using a 26G ½ needle with a 1ml syringe. Mice were initially anesthetized with 3% isoflurane with air/O_2_ mix and animals were kept under anaesthesia with 1.5% isoflurane with air/O_2_ mix during surgery). Monitoring procedure and bioluminescence detection were identical to above described orthotopic injection.

### Animal handling and in vivo ethical statement

Experiments were performed according to the Guide for Care and Use of Laboratory Animals (E67-6-482-21) and the European Directive with approval of the regional ethical committee (Reference AL/55/62/02/13). Mice received food and water ad libitum. Animals were sacrificed using CO_2_. All necessary precautions were taken to minimize pain or discomfort of the animals. General health status was monitored 3 times a week by independent observers. Mice were sacrificed when reaching ethical endpoints.

### Statistics

Statistical analyses were performed using Mann Whitney test (for sample n < 30), Log ranked test for survival analysis and sum-of-squares F test using GraphPad software (Prism, USA). P-values are given in the figure legends, and values of P < 0.05 were considered to be statistically significant. Normal distribution of the values was checked using GraphPad software (Prism, USA). A minimum of three independent experiments including at least triplicates was performed for *in vitro* proliferation assay. For *in vivo* experiment sample size calculation anticipated a therapeutic effect of 20% for a standard deviation of 14% and confidence interval of confidence 95% (Lamorte's Power calculation, University of Boston). Results from two independent in vivo experiments were pooled only if fully comparable (no statistical differences between control groups).

## SUPPLEMENTARY FIGURES AND TABLES


