# Supplementary material for: Inhibition of primary breast tumor growth and metastasis using a neuropilin-1 transmembrane domain interfering peptide
Source: Oncotarget. 2016 Jun 16;7(34):54723–32. doi: 10.18632/oncotarget.10101 (PMC5342376; doi:10.18632/oncotarget.10101)
Supplement: Supplementary file 1 [file oncotarget-07-54723-s001.pdf]

Inhibition of primary breast tumor growth and metastasis using a neuropilin-1 transmembrane domain interfering peptide

SUPPLEMENTARY FIGURES AND TABLES

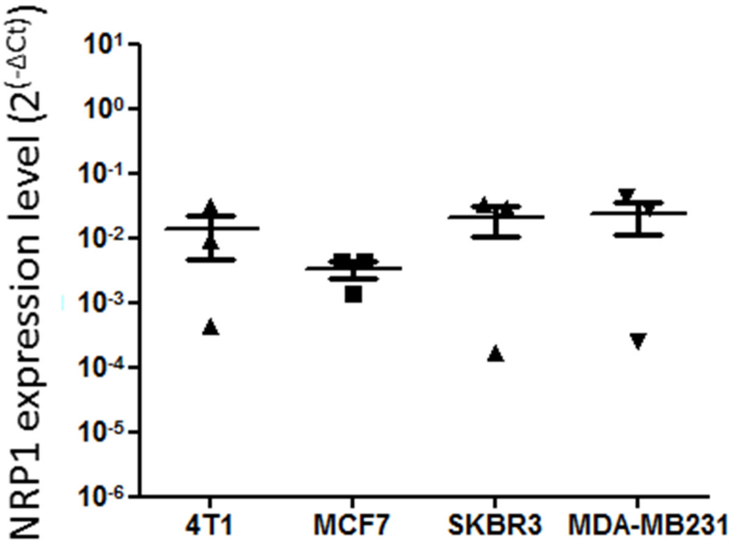

Supplementary Figure S1: expression of NRP1 in the different cell lines. The expression level of NRP1 was determined in 4T1, MCF7, SKBR3 and MDA-MB231 cells using specific primers and RT-QPCR analysis (Applied 7500HT, Sybergreen mix with human h-NRP1_F CCCGAGAGAGCCACTCATG, h-NRP1_R GTCATCACATTCATCCACCAA and mouse mNRP1_F GAGGAATGTTCTGTCGCTATGA, mNRP1_R CCAATGTGAGGGCCAACT primers). The results are presented for three different RNA extracts for each cell lines compared to housekeeping gene GADPH ($2^{-\Delta Ct}$). This analysis revealed similar levels of NRP1 expression in all cell lines consistently with the results obtained at the protein level (see figure 1).

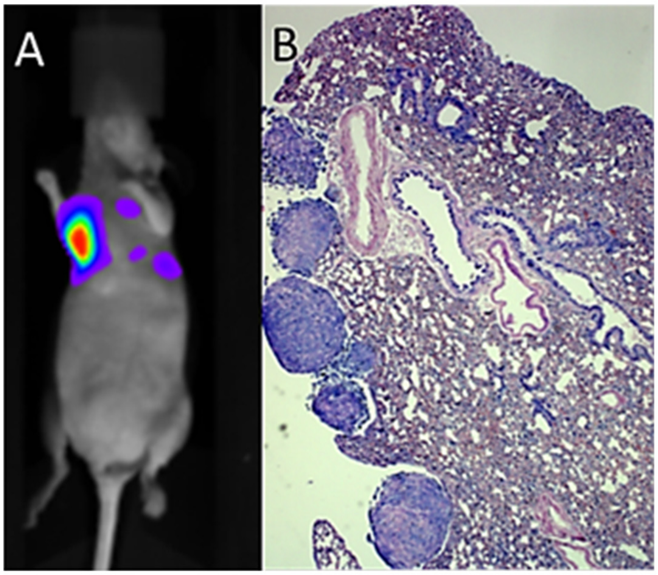

Supplementary Figure S2: histological validation of bioluminescence signals. Quality control experiments were conducted to validate the anatomical concordance of bioluminescent signals and presence of detectable metastases at the histological level. **A.** Image of mouse presenting bioluminescent spots including a large one in the lung. **B.** Histological section (cresyl violet staining) confirming the presence a large multifocal metastasis in the lung.

Supplementary Table S1: Toxicology analysis (blood biochemistry). LDH: Lactate dehydrogenase; ASAT: aspartate transaminase; ALAT alanine transaminase; ALP alkaline phosphatase; creat: creatinine. ns: not significant, ** $p < 0.01$ using mann whitney test.

ANOVA/Bonferroni's Test	Mean Diff.	T	P < 0.05	Summary
LDH cont vs LDH NRP1	261,7	3,308	Yes	**
ASAT cont vs ASAT NRP1	140,5	2,649	No	ns
ALAT cont vs ALAT NRP1	-5,859	0,1105	No	ns
ALP cont vs ALP NRP1	2,222	0,03997	No	ns
Creat cont vs Creat NRP1	-0,8238	0,01611	No	ns
Albumin cont vs Albumin NRP1	1,368	0,02674	No	ns
Bilirubin cont vs Bilirubin NRP1	-0,7233	0,01188	No	ns

Biochemistry analysis of blood samples of mice treated with vehicle (con) or MTP-NRP1 (NRP1). LDH: Lactate dehydrogenase; ASAT: aspartate transaminase; ALAT alanine transaminase; ALP alkaline phosphatase; Creat: creatinine.

Supplementary Table S2: Toxicology analysis (blood cells numeration). HGB: haemoglobin; HCT: hematocrite; PLT: platelets; baso: basophil; Eosino: eosinophil; Mono: monocyte; lympho: lymphocyte. ns: not significant, * $p < 0.05$, mann whitney test.

ANOVA/Bonferroni's Test	Mean Diff.	T	P < 0.05	Summary
HGB cont vs HGB NRP1	-0,6	0,03819	No	ns
Mean Corpuscular Vol cont vs MCV NRP1	3,5	0,2228	No	ns
HCT cont vs HCT NRP1	-2,18	0,1387	No	ns
Plt cont vs Plt NRP1	-51,8	3,297	Yes	*
% Baso cont vs % Baso NRP1	0,11	0,007001	No	ns
% Large Cells cont vs % Large cells NRP1	-0,66	0,042	No	ns
% Eosino cont vs % Eosino NRP1	1,63	0,1037	No	ns
% Mono cont vs % Mono NRP1	1,31	0,08337	No	ns
% Lympho cont vs % Lympho NRP1	-31,37	1,997	No	ns

Numeration analysis of blood samples of mice treated with vehicle (cont) or MTP-NRP1 (NRP1). HGB: haemoglobin; HCT: hematocrite; PLT: platelets; Baso: basophil; Eosino: eosinophil; Mono: monocyte; lympho: lymphocyte.
